# Antioxidant and Anticancer Mechanisms of Unique Polyphenols in *Camellia ptilophylla*: Focus on Gallocatechin-3,5-di-O-gallate and 1,2,4,6-Tetra-O-galloyl-β-D-glucopyranose

**DOI:** 10.3390/molecules30091919

**Published:** 2025-04-25

**Authors:** Langhua Zhou, Sen Lu, Xiong Gao, Zhongzheng Chen, Yuanyuan Zhang, Weixia Zhong, Fuming Zhu, Bin Li, Xiaorong Lin

**Affiliations:** 1 College of Food Science, Scientific Research Base of Tea Comprehensive Utilization Technology Integration of Ministry of Agriculture and Rural Affairs, South China Agricultural University, Guangzhou 510642, China; langhuazhou@126.com (L.Z.); 17079454008@163.com (S.L.); zhongzhengch@scau.edu.cn (Z.C.); zhangyy@scau.edu.cn (Y.Z.); 17817117860@163.com (W.Z.); zhu163fuming@163.com (F.Z.); 2 Institute of Food Microstructure, College of Food and Bioengineering, Fujian Polytechnic Normal University, Fuqing 350300, China; gaoxiong881109@163.com; 3 Guangdong Provincial Key Laboratory of Nutraceuticals and Functional Foods, Guangzhou 510642, China

**Keywords:** *Camellia ptilophylla*, GC-3,5-diGA, 1,2,4,6-GA-glc, antioxidant, anticancer

## Abstract

*Camellia ptilophylla* Chang (*C. ptilophylla*), a unique low-caffeine tea species, is valued for its bioactive properties, especially antioxidant and anticancer activities, due to its distinct phytochemical profile. However, its precise constituents and mechanisms remain poorly understood. This study employs an integrated approach combining chromatographic separation, bioinformatic analysis, and cellular assays to systematically investigate the antioxidant and anticancer properties of *C. ptilophylla* and elucidate its underlying molecular mechanisms. Quantitative analysis revealed that in addition to *trans*-catechins, the unique polyphenolic compounds, gallocatechin-3,5-di-O-gallate (GC-3,5-diGA) and 1,2,4,6-tetra-O-galloyl-β-D-glucopyranose (1,2,4,6-GA-glc), constituted significant proportions of *C. ptilophylla* extracts, with concentrations of 10.25 ± 0.29% and 6.60 ± 0.14%, respectively. Monomeric activity assessment demonstrated that both GC-3,5-diGA and 1,2,4,6-GA-glc exhibited pronounced antiproliferative effects against three cancer cell lines including the Lymph Node Carcinoma of the Prostate cell, human colon cancer cell, and human breast cancer cell. Notably, these compounds demonstrated potent antioxidant capacity, with 62.5 μM of GC-3,5-diGA and 15.63 μM of 1,2,4,6-GA-glc protecting against tBHP-induced oxidative stress in NIH3T3 cells comparable to 125 μM of epigallocatechin gallate and gallocatechin gallate in half-maximal inhibitory concentration. Mechanistic studies revealed that these polyphenols modulated antioxidant defenses and reactive oxygen species homeostasis via targets like fibroblast growth factor 2, telomerase reverse transcriptase, matrix metalloproteinase 9, and ATP-binding cassette subfamily G member 2, inducing oxidative stress and mitochondrial apoptosis to inhibit carcinogenesis. These findings enhance our understanding of the bioactive components responsible for the anticancer and antioxidant properties of *C. ptilophylla* and provide a scientific basis for the development of this dual-purpose plant for food and medicinal applications.

## 1. Introduction

Oxidative stress, resulting from an imbalance between the production and elimination of reactive oxygen species (ROS), plays a pivotal role in the initiation and progression of cancer [1]. Through multiple mechanisms, oxidative stress significantly contributes to tumorigenesis and cancer development. These mechanisms include deoxyribonucleic acid (DNA) damage and mutations, the activation of signaling pathways, the promotion of inflammatory responses, the inhibition of apoptosis, the stimulation of angiogenesis, the modification of the extracellular matrix, metabolic reprogramming, and immune evasion [2,3]. Collectively, oxidative stress acts as a driving force at various stages of cancer progression. Consequently, strategies targeting the regulation of oxidative stress hold significant potential for cancer prevention and treatment [4]. Natural compounds have gained considerable attention in the context of oxidative stress-related cancer prevention and therapy due to their multifaceted advantages [5]. These advantages include high safety profiles, multi-target effects, potent antioxidant capabilities, and broad-spectrum bioactivities. Such properties make natural compounds ideal candidates for cancer prevention and adjuvant therapy, while also providing a promising direction for the development of novel anticancer drugs [6].

Plant polyphenols, including tea polyphenols such as epigallocatechin gallate (EGCG) and gallocatechin gallate (GCG), are widely recognized for their potent antioxidant and anticancer properties [7]. These compounds can modulate the redox status of cells depending on their dose, exposure time, and environmental context [8,9]. At low concentrations, plant polyphenols act as antioxidants, scavenging excessive ROS and protecting normal cells from oxidative damage. Conversely, at high concentrations, they serve as prooxidants, inducing toxicity in cancer cells by enhancing ROS production, to which cancer cells are particularly sensitive [8,9,10,11]. This dual role makes plant polyphenols versatile agents in both disease prevention and cancer therapy.

*Camellia ptilophylla* Chang (*C. ptilophylla*), a naturally occurring low-caffeine (CAF) tea plant, was discovered in the Longmen region of southern China by Professor Chang in 1981 [12,13]. It provides health benefits without the CAF-related side effects such as insomnia and anxiety [12], including superior antioxidant [13], anti-inflammatory [14], anti-obesity [15], and anticancer activities [16,17], compared to the widely cultivated *Camellia sinensis* (*C. sinensis*). The distinctive composition of tea polyphenols in *C. ptilophylla* is responsible for its significant health-promoting properties. Unlike common tea varieties, which are rich in *cis*-catechins such as epigallocatechin gallate (EGCG), *C. ptilophylla* is characterized by a high content of *trans*-catechins, including gallocatechin gallate (GCG), as well as rare polyphenols such as 1,2,4,6-tetra-O-galloyl-β-D-glucopyranose (1,2,4,6-GA-glc), gallocatechin-3,5-di-O-gallate (GC-3,5-diGA), and a novel proanthocyanidin dimer GC-(4→8)-GCG [18,19]. These polyphenols are rich in ortho-dihydroxy groups (catechol structures), a structural feature that likely enhances their biological activities [20,21]. Notably, our previous studies have demonstrated that GC-3,5-diGA and 1,2,4,6-GA-glc exhibit stronger antioxidant activities than GCG [22], although the underlying mechanisms remain unveiled.

This study integrated ultra-high-performance liquid chromatography–quadrupole-time-of-flight tandem mass spectrometry (UPLC-Q-TOF-MS/MS) and high-performance liquid chromatography (HPLC), network pharmacology, and cellular assays to evaluate the antiproliferative activity and oxidative stress protection capabilities of *C. ptilophylla*. The primary bioactive components were identified and analyzed, particularly focusing on GC-3,5-diGA and 1,2,4,6-GA-glc. Using network pharmacology, we systematically explored the core targets, signaling pathways, and biological processes involved in their anticancer and antioxidant effects. Cellular experiments were conducted to further validate their antiproliferative activity against cancer cells and their mechanisms of protection against oxidative stress-induced damage. This study enhances the understanding of *C. ptilophylla* and its specific bioactive components in mediating anticancer and antioxidative activities and provides a scientific foundation for the development of this dual-purpose food and medicine plant.

## 2. Results

### 2.1. Oxidative Stress Pathways Involved in Three Major Cancers

**Figure 1:**
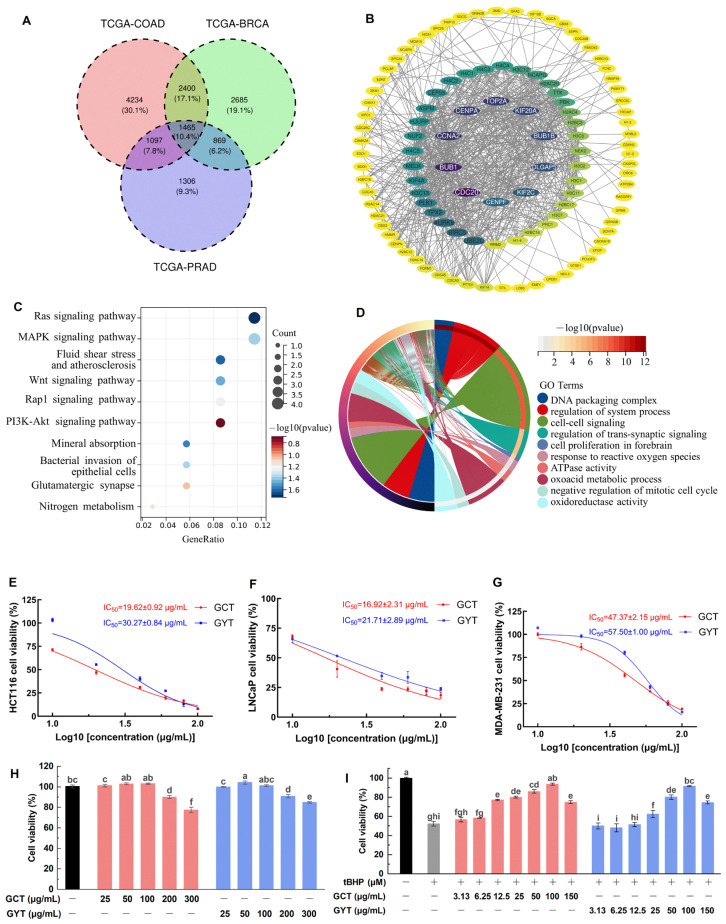
Differential gene expression and functional enrichment analysis of a Venn diagram depicting 1465 common target genes shared between BRCA, COAD, and PRAD (**A**); PPI network analysis of shared DEGs (**B**); KEGG pathway analysis identifying shared pathways between BRCA, COAD, and PRAD (**C**); GO enrichment analysis highlighting key biological processes associated with BRCA, COAD, and PRAD (**D**). Dose–response curves of GCT and GYT demonstrating antiproliferative effects against HCT116 (**E**), LNCaP (**F**), and MDA-MB-231 (**G**) cell lines with corresponding IC_50_ values. Effect of GCT and GYT on the viability of NIH3T3 cells (**H**). Effects of GCT and GYT on the viability of tBHP-induced NIH3T3 cells (**I**). Different letters (a–i) above bars indicate statistically significant differences (*p* < 0.05) among different treatment groups.

Given the worldwide prevalence of colorectal adenocarcinoma (COAD), prostate adenocarcinoma (PRAD), and breast cancer (BRCA), and the firmly established link between oxidative stress and carcinogenesis, we sought to understand the broader implications of oxidative stress pathways in these three major cancers by network pharmacology. As shown in Figure 1A, differential expression analysis across these cancers revealed 1465 commonly dysregulated genes. Protein–protein interaction (PPI) network analysis of these shared differentially expressed genes (DEGs) revealed several highly connected hub genes (Figure 1B), indicating their potential central role in coordinating cellular responses to oxidative stress. The Kyoto Encyclopedia of Genes and Genomes (KEGG) pathway analysis (Figure 1C) predicted that these shared DEGs were significantly enriched in critical signaling cascades, including the Ras, mitogen-activated protein kinase (MAPK), and phosphoinositide 3-kinase-protein kinase B (PI3K-Akt) pathways. These pathways are known to be involved in modulating cellular responses to oxidative stress and interact with protective mechanisms against oxidative damage. The analysis also identified pathways involved in the bacterial invasion of epithelial cells and nitrogen metabolism, suggesting broader implications for cellular homeostasis. The Gene Ontology (GO) enrichment analysis (Figure 1D) revealed significant associations between shared DEGs and biological processes including DNA packaging, system process regulation, and cell–cell signaling.

### 2.2. Cellular Protection Against Oxidative Stress and Anticancer Activity of Tea Extracts

Based on the properties related to oxidative stress and cancer elucidated through network pharmacology analysis, the activities of green tea alcohol extracts from *C. ptilophylla* (GCT) and *C. sinensis* (GYT) (a typical representative tea) in anticancer cell proliferation and antioxidative stress damage were investigated using the methyl thiazolyl tetrazolium (MTT) assay (Figure 1E–G). In the experiment on anticancer cell proliferation, we assessed the inhibitory effects of GCT and GYT on the proliferation of human colon cancer cells (HCT116), Lymph Node Carcinoma of the Prostate cells (LNCaP), and human breast cancer cells (MDA-MB-231). The results indicated that both tea extracts suppressed cancer cell proliferation in a dose-dependent manner. The half-maximal inhibitory concentration (IC_50_) values of GCT for the three cancer cell lines were lower than those of GYT, indicating a stronger anticancer activity of GCT.

Tert-butyl hydroperoxide (tBHP), an oxidizing agent, is commonly used to induce oxidative stress in various cellular systems [23,24]. Mouse embryonic fibroblast cells (NIH3T3) are widely used as a standard model for oxidative stress studies [25,26,27]. In this study, an oxidative stress injury model of NIH3T3 cells induced by tBHP was established to evaluate the antioxidant activities of GCT and GYT. As shown in Figure 1H, GCT and GYT at a concentration of 100 µg/mL did not significantly affect the viability of NIH3T3. Regarding the antioxidative stress damage experiment, the tBHP of 200 µM substantially reduced the viability of NIH3T3 cells to 52.22 ± 2.44% (Figure 1I). However, this reduction was ameliorated by GCT and GYT in a concentration-dependent manner within a safe concentration range. Notably, compared with GYT, GCT manifested stronger antioxidative stress capabilities. Specifically, at a low dosage of 12.5 µg/mL, GCT remarkably enhanced the viability of oxidatively damaged cells. Statistical analysis demonstrated a significant disparity between the GCT-treated group and the oxidative damage group, underscoring GCT’s predominance in alleviating oxidative stress-induced cell impairment.

### 2.3. Major Chemical Compositions of GCT and GYT

The biological activity of tea is highly dependent on its chemical composition. It is well known that tea polyphenols endow tea with excellent antioxidant and anticancer activities. To explore the core components of the antioxidant and anticancer abilities of GCT and GYT, HPLC was used to analyze their main polyphenol components and characteristic alkaloid components.

**Figure 2:**
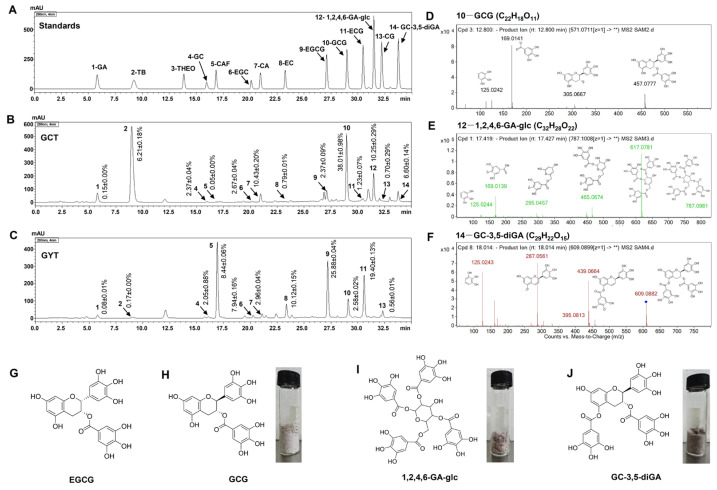
The HPLC chromatograms of standards (**A**), GCT (**B**), and GYT (**C**). ESI-MS^2^ spectra of GCG (**D**), 1,2,4,6-GA-glc (**E**), and GC-3,5-diGA (**F**) in negative ion mode and the chemical structure of EGCG (**G**), GCG (**H**), 1,2,4,6-GA-glc (**I**), and GC-3,5-diGA (**J**). Peak identification: 1-GA, 2-TB, 3-theophylline (THEO), 4-GC, 5-CAF, 6-EGC, 7-CA, 8-EC, 9-EGCG, 10-GCG, 11-ECG, 12-1,2,4,6-GA-glc, 13-CG, and 14- GC-3,5-diGA; GCT—Green tea from *C. ptilophylla*; GYT—green tea from *C. sinensis*. The asterisks (**) in (**D**–**F**) denote representative fragment ions from the Agilent secondary mass spectra.

As shown in Figure 2B, the major components of GCT primarily consist of various *trans*-catechins (components **4**, **7**, **10**, **13**) and theobromine (TB, component **2**). In contrast, GYT is mainly composed of multiple *cis*-catechins (components **6**, **8**, **9**, and **11**) and CAF (component **5**), as shown in Figure 2C. Moreover, GCT contains compound **12** and compound **14**, which are absent in GYT. Mass spectrometry analysis of compounds **10**, **12**, and **14** in GCT identified [M − H]^−^ ions at *m*/*z* values of 457.0777, 787.0981, and 609.0882, respectively, as depicted in Figure 2D–F. These findings align with previous studies [22,28], confirming the identities of these compounds as GCG, 1,2,4,6-GA-glc, and GC-3,5-diGA. The structural configurations and monomeric states of these compounds are presented in Figure 2H–J. In GCT, their relative abundances are 38.01 ± 0.98%, 10.25 ± 0.29%, and 6.60 ± 0.14%, respectively. In addition, the content of EGCG, the primary component of GYT, is 25.88 ± 0.04% (Table S1).

### 2.4. Mechanism Prediction of the Effects of GC-3,5-diGA and 1,2,4,6-GA-glc on Cancers and Oxidative Stress

SwissTargetPrediction [29] was employed to identify the potential targets of GC-3,5-diGA and 1,2,4,6-GA-glc, followed by KEGG pathway analysis. The results indicate that both compounds prominently regulate cancer-associated pathways, including neuroactive ligand–receptor interaction, MAPK signaling, and PI3K-Akt signaling (Figure S1), suggesting their potential as bioactive components targeting oncogenic signaling.

**Figure 3:**
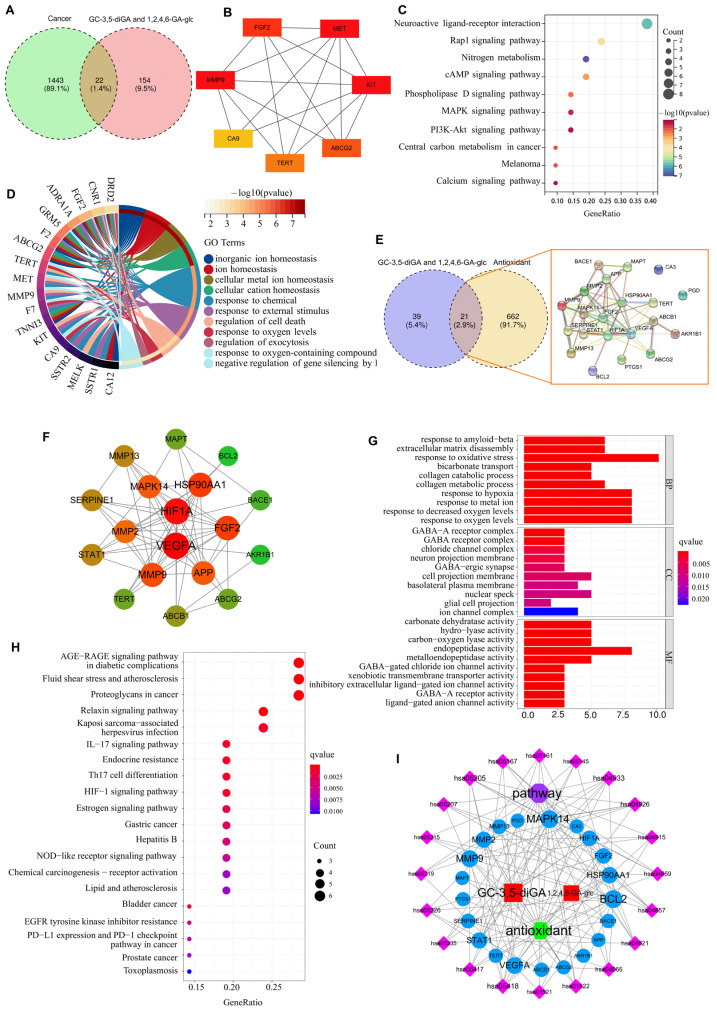
Network pharmacology analysis of GC-3,5-diGA and 1,2,4,6-GA-glc in cancer and oxidative stress. Panels (**A**–**D**) illustrate the analysis of the two compounds in relation to cancer, while panels (**E**–**I**) depict the analysis of the two polyphenols in the context of oxidative stress. (**A**) Venn diagram showing the overlap of genes regulated by the compounds and cancer-associated genes. (**B**) Protein–protein interaction network of hub genes is commonly regulated by both compounds. (**C**) KEGG pathway enrichment analysis of shared target genes. (**D**) GO biological process analysis of target genes. (**E**) Interaction diagram of protein networks at the intersection of two special polyphenols and oxidation. (**F**) The target is highly associated with components shown by the PPI network. Analysis results of GO (**G**) and KEGG (**H**), and (**I**) antioxidant–target–pathway network diagram.

Venn diagram analysis identified 22 overlapping targets between the screened 1443 cancer-related targets and 154 targets of GC-3,5-diGA and 1,2,4,6-GA-glc (Figure 3A). PPI network analysis revealed seven hub genes [fibroblast growth factor 2 (FGF2), MET proto-oncogene (MET), KIT proto-oncogene (KIT), ATP-binding cassette subfamily G member 2 (ABCG2), telomerase reverse transcriptase (TERT), matrix metalloproteinase 9 (MMP9), and carbonic anhydrase 9 (CA9)] that were critically involved in cell proliferation and metastasis (Figure 3B). Further KEGG pathway and GO enrichment analysis demonstrated their significant roles in cellular ion homeostasis, oxidative stress response, and programmed cell death regulation (Figure 3C,D). The analysis of oxidative stress identified 21 common targets between GC-3,5-diGA and 1,2,4,6-GA-glc, two polyphenolic compounds, and pathways related to oxidative stress (Figure 3E). Key targets such as hypoxia-inducible factor 1 subunit alpha (HIF1A), vascular endothelial growth factor A (VEGFA), FGF2, MMP9, and B-cell lymphoma 2 (BCL2) (Figure 3F) were found to play crucial roles in mediating oxidative stress responses and cellular protection. According to GO enrichment analysis (Figure 3G), these targets are predominantly associated with biological processes, including the response to oxidative stress, hypoxia, and decreased oxygen levels. Additionally, KEGG pathway enrichment analysis highlighted that pathways such as the advanced glycation end products–receptor for advanced glycation end products (AGE-RAGE) signaling pathway in diabetic complications, fluid shear stress and atherosclerosis, and proteoglycans in cancer are significantly linked to the regulation of oxidative stress (Figure 3H).

### 2.5. Molecular Docking of GC-3,5-diGA and 1,2,4,6-GA-glc with Shared Targets Related to Cancer and Oxidative Stress

To investigate the interaction modes and binding affinities of GC-3,5-diGA and 1,2,4,6-GA-glc with shared targets involved in cancer and oxidative stress, we conducted systematic molecular docking analyses on four target proteins: FGF2, TERT, MMP9, and ABCG2. For comparative analysis, Ko 143 (a known ABCG2 inhibitor) and MMP9-IN-1 (a selective MMP9 inhibitor) were employed as positive controls. It should be noted that for the comparative docking studies regarding the inhibition or activation of FGF2 and TERT, there were no suitable positive controls available.

**Figure 4:**
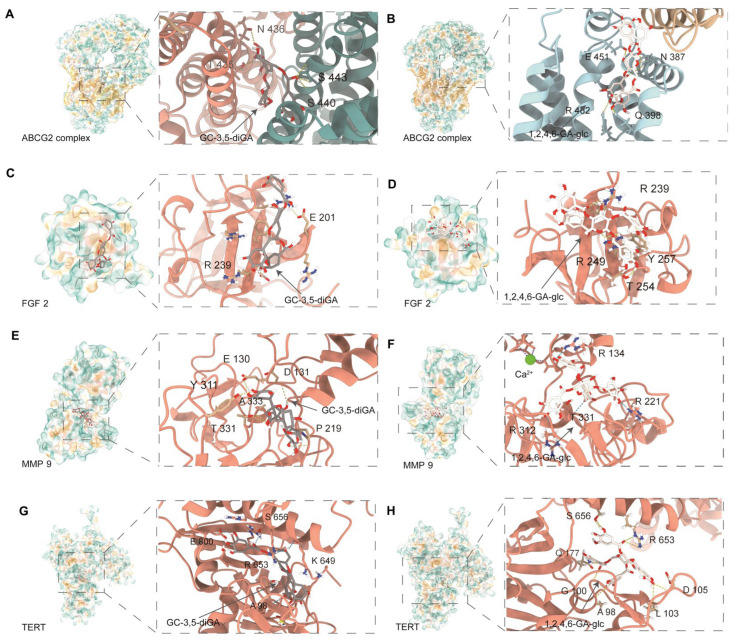
Molecular docking of GC-3,5-diGA and 1,2,4,6-GA-glc with ABCG2, FGF2, MMP9, and TERT. Interaction diagrams between (**A**) ABCG2 and GC-3,5-diGA; (**B**) ABCG2 and 1,2,4,6-GA-glc, (**C**) FGF2 and GC-3,5-diGA; (**D**) FGF2 and 1,2,4,6-GA-glc; (**E**) MMP9 and GC-3,5-diGA; (**F**) MMP9 and 1,2,4,6-GA-glc, the green dot indicates calcium ions (Ca^2+^) in MMP9 protein; (**G**) TERT and GC-3,5-diGA, and (**H**) TERT and 1,2,4,6-GA-glc.

As shown in Figure 4, GC-3,5-diGA and 1,2,4,6-GA-glc exhibited strong binding interactions within the active sites of these proteins through multiple hydrogen bonds and hydrophobic interactions. The docking binding energies of both polyphenols with the targets were less than −5 kcal/mol. The binding affinities of the two polyphenols for ABCG2 and MMP9 were the strongest, followed by TERT and FGF2. Specifically, the binding energies of GC-3,5-diGA and 1,2,4,6-GA-glc with ABCG2 were −10.7 kcal/mol and −9.1 kcal/mol, respectively; −10.4 kcal/mol and −10.6 kcal/mol with MMP9, respectively; −9.5 kcal/mol and −9.3 kcal/mol with TERT, respectively; −7.9 kcal/mol and −7.5 kcal/mol with FGF2, respectively. Molecular docking results for positive controls (Figure S2, Table S2) indicate that GC-3,5-diGA and 1,2,4,6-GA-glc bind to ABCG2 with affinities similar to Ko 143 (−9.3 kcal/mol), and both polyphenols display stronger predicted binding to MMP9 than MMP9-IN-1 (−8.8 kcal/mol). Their results suggest their potential as ABCG2- and MMP9-targeting agents. Given the dual roles of these targets in cancer progression and oxidative stress regulation, these findings provide potential molecular mechanisms underlying the anticancer and antioxidant effects of GC-3,5-diGA and 1,2,4,6-GA-glc.

### 2.6. Cellular Protection Against Oxidative Stress and Anticancer Activities of GC-3,5-diGA and 1,2,4,6-GA-glc

**Figure 5:**
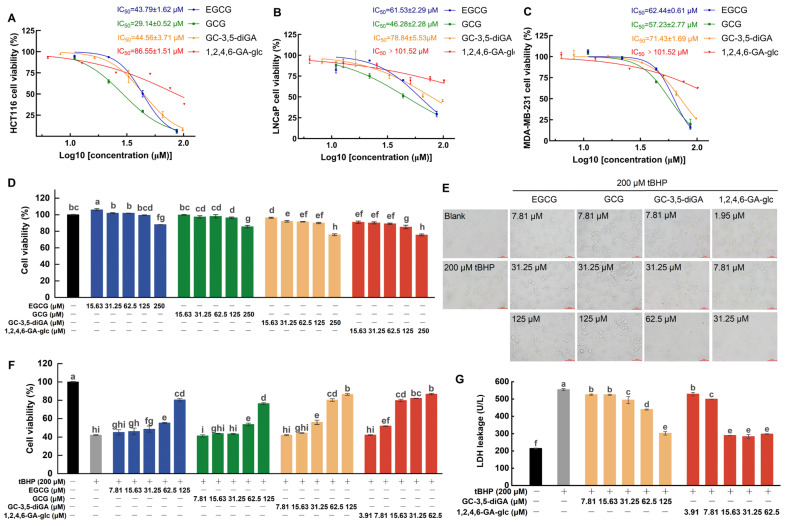
Antiproliferative and antioxidant effects of GC-3,5-diGA and 1,2,4,6-GA-glc on cancer cells. Dose–response curves of four tea polyphenol monomers demonstrating antiproliferative effects against HCT116 (**A**), LNCaP (**B**), and MDA-MB-231 (**C**) cell lines with corresponding IC_50_ values. The effect of four tea polyphenols on the viability of NIH3T3 cells (**D**). The effects of four tea polyphenols on cell morphology (400×) (**E**), cell viability (**F**), and the activity of LDH (**G**) in NIH3T3 cells induced by tBHP. The four tea polyphenols referred to are EGCG, GCG, GC-3,5-diGA, and 1,2,4,6-GA-glc. Different letters (a–i) above bars indicate statistically significant differences (*p* < 0.05) among different treatment groups.

To evaluate the contributions of GC-3,5-diGA and 1,2,4,6-GA-glc to the anticancer and antioxidant activities of GCT, this study assessed the inhibitory effects of GC-3,5-diGA and 1,2,4,6-GA-glc on the proliferation of three human cancer cell lines (HCT116, LNCaP, and MDA-MB-231) using the MTT assay, as well as their effects on cell proliferation under oxidative stress, using EGCG and GCG as controls. As depicted in Figure 5A–C, EGCG, GCG, GC-3,5-diGA, and 1,2,4,6-GA-glc suppressed cancer cell proliferation in a dose-dependent manner. Among them, GCG exhibited the strongest antiproliferative activity against three cancer cell lines, followed by EGCG, GC-3,5-diGA, and 1,2,4,6-GA-glc. The IC_50_ values of EGCG, GCG, GC-3,5-diGA, and 1,2,4,6-GA-glc for HCT116 cells were significantly lower than those for the other two cancer cells, indicating their stronger inhibitory effects on HCT116 cell proliferation than those on LNCaP and MDA-MB-231 cells.

Figure 5D illustrates the effects of EGCG, GCG, GC-3,5-diGA, and 1,2,4,6-GA-glc on the viability of NIH3T3 cells. NIH3T3 cell viability is found at or above 90% upon treatment with EGCG, GCG, or GC-3,5-diGA across concentrations ranging from 0 to 125 μM. Similarly, 1,2,4,6-GA-glc preserved cell viability at or above this threshold with concentrations from 0 to 62.5 μM. These results demonstrate that EGCG, GCG, GC-3,5-diGA, and 1,2,4,6-GA-glc exhibit negligible cytotoxicity in normal NIH3T3 cells at these concentrations.

To investigate the protective effects of four polyphenols against tBHP-induced oxidative stress in NIH3T3 cells, cell proliferation and membrane integrity were evaluated using the MTT assay and lactate dehydrogenase (LDH) leakage assay, respectively, while cell morphology was examined under a microscope. The exposure of NIH3T3 cells to 200 μM of tBHP induced oxidative stress, leading to a significant reduction in cell viability (Figure 5F), a morphological shift from the normal adherent spindle-shaped phenotype to a rounded and partially detached state (Figure 5E), and compromised membrane integrity, as indicated by the increased leakage of intracellular LDH into the culture medium (Figure 5G). Co-treatment with the four polyphenols significantly mitigated the oxidative damage induced by tBHP, with 1,2,4,6-GA-glc demonstrating the most pronounced protective effect. The potent antioxidant activity of 1,2,4,6-GA-glc was evidenced by its ability to significantly enhance cell viability at a low concentration (15.63 μM), achieving a level of protection comparable to that of 125 μM of EGCG or GCG and 62.5 μM of GC-3,5-diGA (Figure 5F). Furthermore, 15.63 μM of 1,2,4,6-GA-glc effectively reduced LDH leakage to a minimal level, preserving membrane integrity and preventing LDH release (Figure 5G). As illustrated in Figure 5E, at equivalent concentrations, 1,2,4,6-GA-glc provided superior protection of cell morphology compared to EGCG, GCG, and GC-3,5-diGA. Specifically, cells treated with 1,2,4,6-GA-glc at 31.25 μM exhibited normal adherent growth and typical fibroblast morphology, closely resembling the untreated control cells. By comparison, cells treated with 31.25 μM of GC-3,5-diGA showed partial protection, with a subset of cells maintaining fibroblast morphology, although signs of damage were still evident. When the concentration of GC-3,5-diGA was increased to 62.5 μM, it significantly protected cell morphology, with cells maintaining adherent fibroblast morphology and only minimal signs of damage observed. However, EGCG and GCG treatments provided limited protection, as evidenced by a substantial proportion of damaged cells remaining.

These findings demonstrate that the antioxidant capacities of GC-3,5-diGA and 1,2,4,6-GA-glc are significantly higher than those of EGCG and GCG, highlighting their potential as effective antioxidants in mitigating oxidative stress-induced cellular damage.

### 2.7. Cellular Protection Mechanisms Against Oxidative Stress Mediated by GC-3,5-diGA and 1,2,4,6-GA-glc

Antioxidants confer cellular protection through integrated mechanisms: the activation of endogenous enzymes (superoxide dismutase, SOD; catalase, CAT; glutathione peroxidase, GSH-Px) and the enhancement of non-enzymatic systems (reduced glutathione, GSH), direct free radical scavenging and generation inhibition, and the modulation of redox-sensitive signaling pathways. These coordinated actions enhance cellular antioxidant capacity and mitigate oxidative damage while maintaining physiological function [30,31].

**Figure 6:**
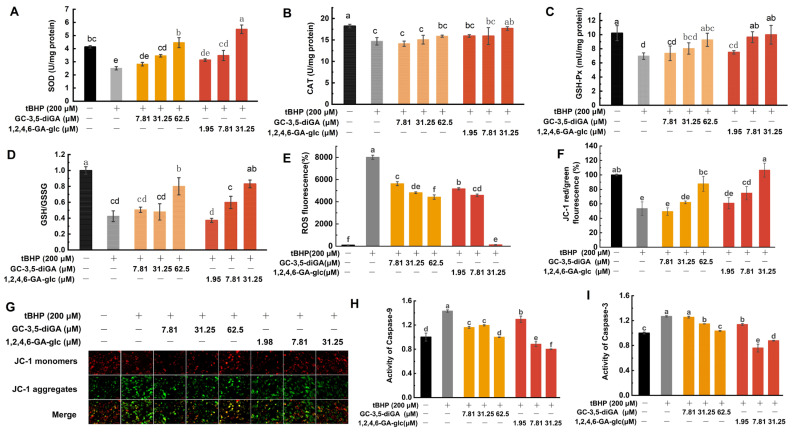
Protective mechanisms of GC-3,5-diGA and 1,2,4,6-GA-glc against tBHP-induced oxidative stress in NIH3T3 cells. Activities of antioxidant enzymes SOD (**A**), CAT (**B**), and GSH-Px (**C**); GSH/GSSG ratio (**D**); ROS levels (**E**); mitochondrial membrane potential (MMP) (**F**); MMP fluorescence images (**G**); activities of caspase-9 (**H**) and caspase-3 (**I**). Different letters (a–f) above bars indicate statistically significant differences (*p* < 0.05) among different treatment groups.

In this study, the activities of SOD, CAT, and GSH-Px, as well as the levels of GSH and oxidized glutathione (GSSG), were analyzed. As shown in Figure 6A–D, tBHP-induced oxidative damage in NIH3T3 cells resulted in a significant reduction in the activities of SOD, CAT, and GSH-Px, accompanied by the conversion of intracellular GSH to GSSG, leading to a reduced GSH/GSSG ratio. A decline in the GSH/GSSG ratio indicates an elevated level of cellular oxidative damage [32]. However, treatment with GC-3,5-diGA and 1,2,4,6-GA-glc significantly enhanced the activities of these endogenous antioxidant enzymes and increased the GSH/GSSG ratio in a dose-dependent manner.

Oxidative damage-generated ROS disrupts the lipids and proteins of mitochondrial membranes, leading to increased membrane permeability. ROS also depletes antioxidants such as GSH, exacerbating the reduction in mitochondrial membrane potential (MMP). The decrease in MMP is an early marker of cellular damage and apoptosis. During apoptosis, the reduction in MMP activates cysteine-dependent aspartate-specific protease 9 (caspase-9), initiating the apoptotic program, while the activation of cysteine-dependent aspartate-specific protease 3 (caspase-3) marks the irreversible execution phase of apoptosis [31]. To assess the effects of tBHP-induced oxidative stress, intracellular ROS levels were assessed using a DCFH-DA fluorescence probe. MMP changes were analyzed using an MMP assay kit with JC-1, and caspase activity was measured using a colorimetric assay kit. The results showed that tBHP treatment significantly increased ROS levels (Figure 6E) and decreased MMP (Figure 6F,G), subsequently activating caspase-9 (Figure 6H) and caspase-3 (Figure 6I), thereby initiating the apoptotic program. Notably, treatment with GC-3,5-diGA and 1,2,4,6-GA-glc significantly inhibited these effects, thereby attenuating apoptosis. Of particular interest, 1,2,4,6-GA-glc at a high dose of 31.25 μM significantly reduced ROS levels to near baseline values, comparable to those in normal cells.

These findings indicate that GC-3,5-diGA and 1,2,4,6-GA-glc effectively mitigate tBHP-induced oxidative damage in NIH3T3 cells by activating the enzymatic antioxidant mechanism, upregulating GSH, reducing ROS accumulation, maintaining normal MMP, and inhibiting the mitochondrial apoptotic pathway.

## 3. Discussion

Network pharmacology-based analysis of differential expression in three highly prevalent cancers—BRCA, COAD, and PRAD—revealed that oxidative stress is a central driver of cancer pathogenesis, exerting its effects through the modulation of multiple key signaling cascades and biological processes. Specifically, several well-characterized oncogenic pathways, including the PI3K-Akt, MAPK, and Ras pathways, were identified as critical regulators of cell proliferation, survival, and apoptotic signaling. The biological process response to reactive oxygen species unequivocally underscores the pivotal role of oxidative stress in cancer biology. Moreover, functional annotations, such as oxidoreductase activity and the negative regulation of the mitotic cell cycle, provide mechanistic evidence for how oxidative stress fine-tunes redox homeostasis and cell cycle dynamics to drive cancer progression. These findings underscore the critical role of oxidative stress regulation in cancer prevention and treatment. Implementing strategies to prevent oxidative stress before cancer initiation and to neutralize the deleterious impact of elevated oxidative stress during cancer progression represents a pivotal approach in modern cancer prevention and therapeutic development [33,34,35,36].

Tea, a dual-purpose medicinal and edible resource, demonstrates remarkable potential for anticancer and antioxidant effects, largely due to its polyphenolic constituents, which serve as the primary bioactive components responsible for these health-promoting properties [37,38]. As a distinctive tea resource characterized by its low CAF content and high levels of *trans*-catechins, *C. ptilophylla* has garnered significant attention for its robust antioxidant, anti-inflammatory, and anticancer bioactive properties [14,38]. Peng et al. [13] performed an in-depth analysis of the chemical composition and antioxidant activities of various teas, demonstrating that *C. ptilophylla* possesses a markedly superior antioxidant capacity relative to other teas examined in the study. Gao et al. [16] further elucidated the anticancer activities and underlying mechanisms of *C. ptilophylla*. In alignment with previous reports, our results confirm that the antioxidant and anticancer activities of *C. ptilophylla* extract are significantly more potent than those of *C. sinensis*.

The remarkable antioxidant and anticancer advantages of *C. ptilophylla* are attributed to its unique polyphenolic composition, predominantly consisting of *trans*-catechins, which collectively account for 51.51% of the extract. Additionally, GC-3,5-diGA and 1,2,4,6-GA-glc collectively represent 16.85% of the extract. Among these polyphenolic compounds, GCG demonstrates the most pronounced inhibitory effects on cancer cell proliferation, surpassing EGCG, GC-3,5-diGA, and 1,2,4,6-GA-glc. In contrast, GC-3,5-diGA and 1,2,4,6-GA-glc exhibit superior antioxidant activities. Specifically, 1,2,4,6-GA-glc significantly mitigates oxidative stress-induced membrane damage and reduces cell death at a low concentration of 15.63 μM. Similarly, GC-3,5-diGA displays excellent protective effects against oxidative stress at a low concentration of 62.5 μM. In comparison, EGCG and GCG require higher concentrations (125 μM) to exert comparable protective effects against oxidative damage.

The GCT polyphenol–targets–antioxidant/anticancer network analysis demonstrated that GC-3,5-diGA and 1,2,4,6-GA-glc exhibited high degree values, with shared targets including FGF2, TERT, MMP9, and ABCG2. The molecular docking results indicated high binding affinity and potential bioactivity between these four targets and the two polyphenols. In cancer, FGF2 promotes tumor angiogenesis and cell proliferation, while under oxidative stress conditions, it enhances the cellular antioxidant defense by upregulating the expression of antioxidant enzymes (SOD, CAT, and GSH-Px) and maintaining GSH levels [39,40]. TERT regulates mitochondrial function and antioxidant gene expression and reduces ROS production through non-telomerase-dependent mechanisms [41]. MMP9, a key regulator of extracellular matrix components, is associated with cancer and inflammation [42]. Lewins et al. [43] found that MMP9 is involved in cell proliferation and S-phase cell cycle arrest in HCT116 cells. ABCG2 is a transporter protein that pumps out drug compounds, interfering with the effectiveness of drugs during cancer treatment and preventive therapy [44]. Additionally, BCL-2, which is also present in the antioxidant target intersection, plays a crucial role in regulating apoptosis and oxidative stress in cancer cells. Gao et al. [16] reported that cocoa tea inhibits HCT116 proliferation through BCL-2-mediated mitochondrial apoptosis. A pathway analysis of antioxidant-related targets indicated that the response to oxidative stress is significantly enriched in biological processes, while KEGG pathway enrichment highlighted key pathways such as proteoglycans in cancer, the hypoxia-inducible factor-1 signaling pathway, and the mitochondrial apoptosis pathway, which play crucial roles in cancer prevention and treatment. These findings suggest that GC-3,5-diGA and 1,2,4,6-GA-glc from *C. ptilophylla* may exert their anticancer effects through interactions with target proteins and multiple signaling pathways, primarily by leveraging their antioxidant properties. Specifically, they exhibit significant regulatory effects on intracellular antioxidant enzymes and ROS levels, as validated by our cellular experiments.

The antioxidant and anticancer activities of polyphenols are closely related to their structural features, including the number and position of the hydroxyl groups, the presence of conjugated systems, and stereochemical configurations [20,21,45,46,47,48]. EGCG, a prototypical *cis*-catechin polyphenol, exhibits potent antioxidant and anticancer activities. Its structure comprises two benzene rings (A- and B-rings) and a pyran ring (C-ring), with three adjacent hydroxyls on the B-ring, two hydroxyls on the A-ring, and a galloyl group attached to the C-ring [49]. The presence of eight phenolic hydroxyls in EGCG enables the simultaneous scavenging of multiple free radicals by hydrogen donation, effectively terminating radical chain reactions [45]. Moreover, the conjugated system (comprising C=C double bonds, benzene rings, and the galloyl moiety linked via an ester bond) enhances radical trapping efficiency [46,48,50,51]. This mechanism not only maintains normal cellular redox homeostasis but also suppresses oxidative stress-mediated carcinogenesis [10,34]. Notably, GCG, an EGCG isomer with identical hydroxyl numbers, shows comparable protection against tBHP-induced oxidative damage yet demonstrates superior antiproliferative effects. This enhanced activity likely stems from its optimized spatial arrangement of phenolic hydroxyls, which facilitates tighter binding to cancer cell targets and the more efficient disruption of proliferative signaling [52,53], highlighting the critical role of stereochemistry in bioactivity.

By contrast, GC-3,5-diGA, despite its higher phenolic hydroxyl content, exhibits reduced anticancer activity compared to EGCG and GCG. This diminished efficacy appears attributable to steric hindrance from its additional galloyl group on the A-ring, which compromises target engagement [54]. Similarly, 1,2,4,6-GA-glc, with four gallate groups, shows limited bioactivity potentially due to intracellular transport limitations imposed by its bulky structure. Intriguingly, the strong antioxidant capacity of GC-3,5-diGA and 1,2,4,6-GA-glc may counteract cytotoxic ROS accumulation in cancer cells [54], whereas EGCG and GCG selectively promote apoptosis through ROS induction [55,56]. Although the anticancer activities of GC-3,5-diGA and 1,2,4,6-GA-glc are lower than those of EGCG and GCG, they contain multiple phenolic hydroxyl groups and possess an extended conjugated system, which endows them with a stronger free-radical-scavenging ability. Although these structural features limit their direct cytotoxic effects, they confer potent chemo-preventive benefits through superior antioxidant mechanisms, effectively interfering with cancer initiation and progression pathways.

This study advances our understanding of the bioactive constituents mediating the anticancer and antioxidant properties of *C. ptilophylla*, while establishing a scientific foundation for its development as a potential therapeutic agent with dual applications in cancer chemoprevention and adjuvant therapy.

## 4. Materials and Methods

### 4.1. Materials and Chemical Reagents

Apical buds and two to three adjacent leaves of *C. ptilophylla* (Figure S3) were collected from Nankun Mountain (geographic coordinates: 113°48′35″ E–113°55′00″ E, 23°36′58″ N–23°43′14″ N, Longmen, Guangdong, China) in spring 2019. Dried green tea from *C. ptilophylla* was prepared as previously described by Gao et al. [16]. Yunnan Daye green tea from *C. sinensis* was sourced from Huahai Sugar Development Co., Ltd. (Xuwen, China). Both teas were pulverized and passed through a sieve with mesh sizes ranging from 20 to 30 (0.9 mm to 0.6 mm).

Epicatechin (EC, ≥98%), epicatechin gallate (ECG, ≥98%), epigallocatechin (EGC, ≥98%), EGCG (≥98%), catechin (CA, ≥98%), catechin gallate (CG, ≥98%), gallocatechin (GC, ≥98%), and GCG (≥98%) were procured from Shanghai Yuanye Bio-Technology Co., Ltd. (Shanghai, China). HPLC-grade acetonitrile and methanol were procured from Merck (Darmstadt, Germany), and HPLC-grade trifluoroacetic acid (TFA) was sourced from Shanghai Macklin Biochemical Co., Ltd. (Shanghai, China). Ethyl acetate and 95% ethyl alcohol were acquired from Sinopharm Chemical Reagent Co., Ltd. (Shanghai, China).

NIH3T3, LNCaP, HCT116, and MDA-MB-231 were obtained from the Chinese Academy of Sciences (Shanghai, China). Assay kits for CAT, SOD, GSH-Px, and ROS were supplied by Beyotime Biotechnology (Shanghai, China). Microscale assay kits for GSH/GSSG and LDH were bought from Nanjing Jiancheng Bioengineering Institute (Nanjing, China). Sephadex LH-20, tBHP solution (70%), MTT, and Coomassie brilliant blue G-250 were supplied by Sigma-Aldrich (St. Louis, MO, USA).

Dulbecco’s modified Eagle’s medium (DMEM), RPMI-1640 medium, fetal bovine serum (FBS, Australia Origin), Dulbecco’s phosphate-buffered saline (pH 7.4), trypsin–ethylene diamine tetra-acetic acid solution at a concentration of 0.25% was sourced from Gibco (Life Technologies, Carlsbad, CA, USA). Penicillin/streptomycin was sourced from HyClone (General Electric Healthcare, Uppsala, Sweden). The Pierce bicinchoninic acid (BCA) protein assay kit and enhanced chemiluminescent substrate were purchased from Thermo Scientific (Rockford, IL, USA). Caspase-9 and caspase-3 colorimetric assay kits were obtained from Jiangsu Keygen Biotech Corp., Ltd. (Keygen Biotech, Nanjing, China).

### 4.2. Preparation and Chemical Analysis of Tea Extracts

Ten grams of dried green tea from *C. ptilophylla and C. sinensis* were extracted twice using 100 mL of 30% ethanol in water at 70 °C for 25 min, following the method of Kuang et al. [22] with modifications. The extract was concentrated via rotary evaporation (R-3, BUCHI, Flawil, Switzerland) under vacuum to eliminate ethanol. The residue underwent extraction with ethyl acetate twice. The ethyl acetate fraction was collected, concentrated, and subjected to lyophilization using a freeze dryer (Christ Alpha 1-2 LD plus, Benningen, Germany). The extraction yields of the lyophilized GCT and GYT were 22.5% and 19.3%, respectively. The extracts were stored at −20 °C for subsequent analysis.

The constituents in GCT and GYT were assessed via HPLC as described previously by Ying et al. [28]. The analysis was conducted on a Shimadzu (Tokyo, Japan) LC 2030C 3D plus HPLC system with an Agilent Poroshell 120 Bonus-RP column (4.6 mm × 50 mm, 2.7 µm, Agilent, Santa Clara, CA, USA). The mobile phase was composed of 0.05% TFA in ultrapure water (solvent A) and acetonitrile (solvent B). A gradient elution program was executed at a flow rate of 0.8 mL/min at 30 °C, as detailed below: 0–8 min, 0% B; 8–17 min, 0% B to 9% B; 17–25 min, 9% B to 18% B; 25–30 min, 18% B to 24% B; 30–35 min, 24% B to 28% B, 35–40 min, 28% B to 0% B. Signals were recorded at 280 nm.

### 4.3. Isolation and Identification of GCG, GC-3,5-diGA, and 1,2,4,6-GA-glc from GCT

GCG, GC-3,5-diGA, and 1,2,4,6-GA-glc were isolated from GCT as described by Kuang et al. [22] with some modifications. The ethanol/ethyl acetate extracts were subjected to chromatography on a Sephadex LH-20 column using ethanol concentrations of 55%, 75%, and 95% to isolate the target crude fractions. Then, these fractions were further purified using preparative high-performance liquid chromatography (PHPLC) (LC3000, Beijing Innovation Tong Heng Technology Co., Ltd., Beijing, China) connected to a COSMOSIL 5C18-MS-II column (20 mm I.D × 250 mm, 5 μm) (Nacalai Tesque, Inc., Kyoto, Japan). The PHPLC separation conditions for the mobile phase were as follows: the eluents consisted of water (eluent A) and acetonitrile (eluent B). Gradient elution was performed at a flow rate of 12 mL/min with the following profile: 0–8 min, increasing from 18.5% B to 21.0% B; 8–36 min, increasing from 21.0% B to 40.0% B. Detection was set at 210 nm. Subsequently, GCG, GC-3,5-diGA, and 1,2,4,6-GA-glc were identified using a UPLC Q-TOF-LC/MS/MS (1290-6540B, Agilent, Santa Clara, CA, USA) with an electron spray ionization (ESI) ion source. All data for GCG, GC-3,5-diGA, and 1,2,4,6-GA-glc presented in this study were newly generated from independent replicates using updated analytical protocols.

### 4.4. Difference Expression Analysis and Enrichment Analysis of Cancer-Related Genes

The Cancer Genome Atlas (TCGA) [57] with the BRCA, COAD, and PRAD datasets were downloaded from the TCGA database (https://www.cancer.gov/tcga, accessed on 9 December 2024). Differential expression analysis between the disease and healthy groups was conducted using the DESeq2 package (v 1.48.0) [58] in R. Genes were defined as differentially expressed if they met the criteria of *p* < 0.05 and |log_2_FoldChange| > 1. Subsequently, the intersection of DEGs across the TCGA-BRCA, TCGA-COAD, and TCGA-PRAD datasets was used to identify cancer-related genes. To understand the relevant biological functions and pathways of these cancer-related genes, enrichment analysis was performed using the GO [59] and KEGG [60] databases through the clusterProfiler package, with a significance level of *p* < 0.05.

### 4.5. Analysis of GC-3,5-diGA and 1,2,4,6-GA-glc Targets with Cancer-Related Genes and Antioxidant-Related Genes

SwissTargetPrediction [29] was employed to predict the potential targets of GC-3,5-diGA and 1,2,4,6-GA-glc, whose chemical structures were drawn using ChemDraw Pro 20.0. KEGG pathway analysis was subsequently performed on these predicted targets. The predicted targets were analyzed in conjunction with antioxidant-related targets retrieved from the GeneCards database (https://www.genecards.org/, accessed on 16 December 2024) and cancer-related genes identified from the TCGA-BRCA, TCGA-COAD, and TCGA-PRAD datasets. A Venn diagram was created to visualize the overlapping genes. To further elucidate the interactions among these genes, a PPI network was constructed using the STRING database (https://string-db.org, accessed on 17 December 2024) [61] and then visualized and analyzed with Cytoscape software (v. 3.10.1). Additionally, GO functional enrichment analysis and KEGG pathway analysis were performed using R packages (ClusterProfiler, enrichplot, and org.s.eg.db) with a significance threshold of *p* < 0.05. The compound–target–pathway network was constructed to illustrate the relationships between the compounds and their antioxidant and cancer-related pathways.

### 4.6. Molecular Docking

Ko143 (CAS: 461054-93-3) and MMP9-IN-1 (CAS: 502887-71-0) served as positive controls for the inhibitors of ABCG2 and MMP9, respectively. The structures of Ko 143, MMP9-IN-1, and 1,2,4,6-GA-glc were retrieved from the PubChem database (https://pubchem.ncbi.nlm.nih.gov/, accessed on 4 January 2025), and their 3D structure was generated and energy-minimized using Chem3D software (v20.0.0.41). GC-3,5-diGA was also energy-minimized using Chem3D software. All candidate molecules (Ko 143, MMP9-IN-1, GC-3,5-diGA, and 1,2,4,6-GA-glc) were analyzed for their binding affinities and interaction patterns with the target using Autodock Vina 1.5.7 software. The initial models of FGF2 and TRET were predicted with AlphaFold3 [62]. MMP9 (PDB ID: 1L6J; resolution: 2.50 Å) and the ABCG2 complex (PDB ID: 8QCM; resolution: 2.39 Å) were downloaded from the PDB (http://www.rcsb.org/pdb/home/home.do, accessed on 4 January 2025). During the docking analysis, all protein and ligand files were converted to PDBQT format, with all water molecules removed and polar hydrogen atoms added. The grid box was centered to cover each protein’s structural domain and to accommodate the free movement of the ligand. The grid box was set to 30 Å × 30 Å × 30 Å, with a grid point spacing of 0.05 nm. The molecular docking study was conducted using Autodock Vina 1.5.7 (http://autodock.scripps.edu/, accessed on 5 January 2025).

### 4.7. Cell Culture and Cell Viability Assay

NIH3T3 cells were cultured in Dulbecco’s modified Eagle’s medium (DMEM), while HCT116, LNCap, and MDA-MB-231 cells were grown in RPMI 1640 medium. All media were supplemented with 10% FBS, penicillin (100 units/mL), and streptomycin (100 µg/mL). The cells were incubated at 37 °C with 5% CO_2_.

The antioxidant stress experiment was conducted following a modified protocol based on the report by Liu et al. [26]. NIH3T3 cells were seeded in a 96-well plate at a density of 1 × 10^4^ cells/well. After 24 h of incubation, the cells were treated with various concentrations of samples (GCT, GYT, and four polyphenol monomers) or co-cultured with 200 µM of tBHP, followed by further incubation at 37 °C for 24 h. Cells treated with medium alone served as the blank control. After 24 h of treatment, cell images were captured using a BDS400 inverted biological microscope (Chongqing Optec Instrument, Chongqing, China). Cell culture supernatants were collected, and LDH release was measured using an LDH assay kit. Cell viability in the 96-well plate was simultaneously assessed using the MTT assay.

The experiment to inhibit tumor cell proliferation was performed using a modified protocol based on the method described by Gao et al. [16]. HCT116, LNCaP, and MDA-MB-231 cells were seeded in a 96-well plate at a density of 5 × 10^3^ cells/well and allowed to adhere for 24 h. The cells were then treated with samples (GCT, GYT, and four polyphenol monomers) for 48 h. Cells treated with medium alone served as the blank control. Subsequently, cell viability in the 96-well plate was estimated using the MTT assay. Following incubation with the MTT solution and dissolution with dimethyl sulfoxide as previously described by Gao et al. [16], 150 μL of the solution from each well was transferred to a new 96-well plate, and the absorbance at 550 nm was measured using a VersaMax ELISA Microplate Reader (Molecular Devices, Sunnyvale, CA, USA).

### 4.8. Determination of SOD, CAT, GSH-Px, GSH, and GSSG Levels

NIH3T3 cells were seeded at a density of 6 × 10^5^ cells in 60 mm^2^ culture dishes and incubated for 24 h. Subsequently, the cells were co-treated with 200 µM of tBHP and various concentrations of GC-3,5-diGA and 1,2,4,6-GA-glc for 24 h. The levels of SOD, CAT, GSH-Px, GSH, and GSSG in the cell lysate supernatant were measured using commercial kits. Specifically, the activities of SOD, CAT, and GSH-Px were determined using a total SOD assay kit with WST-8, CAT assay kits, and cellular GSH-Px assay kits, respectively. The contents of GSH and GSSG were measured using a total GSH/GSSG assay kit with a microscale. Results were expressed as units of activity per milligram of protein.

### 4.9. Measurement of Intracellular ROS

NIH3T3 cells were seeded in a 24-well plate at a density of 5 × 10^4^ cells per well and incubated for 24 h. Subsequently, the cells were treated with GC-3,5-diGA and 1,2,4,6-GA-glc and co-cultured with 200 µM of tBHP for 6 h. Intracellular ROS levels were measured using a commercial ROS assay kit. ROS levels were assessed by staining with 10 μM of DCFH-DA for 30 min, followed by fluorescence measurement. A quantitative analysis of fluorescence intensity was performed by processing the fluorescence images acquired using a Carl Zeiss Axio Observer A1 fluorescence microscope (Oberkochen, Germany) with ImageJ software (version 1.53k).

### 4.10. MMP Assay

NIH3T3 cells were treated with GC-3,5-diGA, 1,2,4,6-GA-glc, and 200 µM of tBHP in 60 mm^2^ culture dishes. After 24 h of incubation, the cells were harvested to assess MMP. The collected cells were stained with the JC-1 fluorescent probe and analyzed using a SpectraMax i3x fluorescence plate reader (Molecular Devices, Sunnyvale, CA, USA). The JC-1 monomer was detected at an excitation/emission wavelength of 490/530 nm (green fluorescence), while the JC-1 polymer was detected at 525/590 nm (red fluorescence). The red-to-green fluorescence ratio was then calculated to evaluate mitochondrial depolarization. Fluorescence images were captured using a Carl Zeiss Axio Observer fluorescence microscope (Oberkochen, Germany). The acquired images were processed using ImageJ software (version 1.53k).

### 4.11. Caspase-3 and Caspase-9 Activation Assay

The supernatant lysates of NIH3T3 cells treated with GC-3,5-diGA, 1,2,4,6-GA-glc, and 200 µM of tBHP were obtained, and the protein concentration in cell lysates was standardized using the Bradford protein assay [63]. The activities of caspase-9 and caspase-3 in the cell lysis supernatant were then determined using colorimetric assay kits.

### 4.12. Statistical Analysis

Each experiment included three to four biological replicates, and the results are reported as mean ± standard deviation (S.D.). Statistical analyses were conducted using one-way ANOVA with the Tukey–Kramer post hoc test, performed in SPSS 20.0 for Windows. Data visualization and graphical representations were generated using GraphPad Prism 10.4.1, Origin 8.5, and ImageJ software (version 1.53k).

## 5. Conclusions

In summary, the integration of network pharmacology and experimental validation has elucidated the multifaceted roles of GC-3,5-diGA and 1,2,4,6-GA-glc in modulating oxidative stress and cancer-related pathways. These compounds, derived from *C. ptilophylla*, exhibit potent antioxidant and anticancer activities, making them and *C. ptilophylla* promising candidates for further development in cancer prevention and therapy.

## Data Availability

The original contributions presented in the study are included in the article, further inquiries can be directed to the corresponding author.

## Supplementary Materials

The following supporting information can be downloaded at: https://www.mdpi.com/article/10.3390/molecules30091919/s1, Table S1: Major components of GCT and GYT (%). Table S2: Binding Energy for targets with positive controls. Figure S1: KEGG pathway enrichment analysis of GC-3,5-diGA and 1,2,4,6-GA-glc-associated genes. Figure S2: Molecular docking of Ko143 and MMP-9-IN-1 with ABCG2 and MMP9. (A) ABCG2 and GC-3,5-diGA, (B) MMP9 and MMP-9-IN-1. Figure S3: Images of *C. ptilophylla* were collected from Nankun Mountain in spring 2019.

## Abbreviations

ABCG2, ATP-binding cassette subfamily G member 2; AGEs, advanced glycation end products; Akt, protein kinase B; BCL2, B-cell lymphoma 2; BRCA, breast cancer; CA, catechin; CA9, carbonic anhydrase 9; CAF, caffeine; CAT, catalase; caspase-3, cysteine-dependent aspartate-specific protease 3; caspase-9, cysteine-dependent aspartate-specific protease 9; CG, catechin gallate; COAD, colorectal adenocarcinoma; *C. ptilophylla*, *Camellia ptilophylla* Chang; *C. sinensis*, *Camellia sinensis*; DEGs, differentially expressed genes; DNA, deoxyribonucleic acid; DMEM, Dulbecco’s modified Eagle’s medium; EC, epicatechin; ECG, epicatechin gallate; EGC, epigallocatechin; EGCG, epigallocatechin gallate; FGF2, fibroblast growth factor 2; GC, gallocatechin; GC-3,5-diGA, gallocatechin-3,5-di-O-gallate; GCG, gallocatechin gallate; GCT, green tea alcohol extracts from *Camellia ptilophylla*; GO, Gene Ontology; GSH, reduced glutathione; GSH-Px, glutathione peroxidase; GSSG, oxidized glutathione; GYT, green tea alcohol extracts from *Camellia sinensis*; HCT116, human colon cancer cell; HIF1A, hypoxia-inducible factor 1 subunit alpha; HPLC, high-performance liquid chromatography; IC_50_, half-maximal inhibitory concentration; KEGG, Kyoto Encyclopedia of Genes and Genomes; KIT, KIT proto-oncogene, receptor tyrosine kinase; LDH, lactate dehydrogenase; LNCaP, Lymph Node Carcinoma of the Prostate cell; MAPK, mitogen-activated protein kinase; MDA-MB-231, human breast cancer cell; MET, MET proto-oncogene, receptor tyrosine kinase; MMP, mitochondrial membrane potential; MMP9, matrix metalloproteinase 9; MTT, methyl thiazolyl tetrazolium; NIH3T3, mouse embryonic fibroblast cell; PHPLC, preparative high-performance liquid chromatography; PI3K, phosphoinositide 3-kinase; PPI, protein–protein interaction; PRAD, prostate adenocarcinoma; RAGE, receptor for advanced glycation end products; ROS, reactive oxygen species; SOD, superoxide dismutase; TB, theobromine; tBHP, tert-butyl hydroperoxide; TERT, telomerase reverse transcriptase; TCGA, the Cancer Genome Atlas; TFA, trifluoroacetic acid; UPLC-Q-TOF-MS/MS, ultra-high-performance liquid chromatography–quadrupole-time-of-flight tandem mass spectrometry; VEGFA, vascular endothelial growth factor A; 1,2,4,6-GA-glc, 1,2,4,6-tetra-O-galloyl-β-D-glucopyranose.
