# Supplementary material for: Antioxidant and Anticancer Mechanisms of Unique Polyphenols in Camellia ptilophylla: Focus on Gallocatechin-3,5-di-O-gallate and 1,2,4,6-Tetra-O-galloyl-β-D-glucopyranose
Source: Molecules. 2025 Apr 25;30(9):1919. doi: 10.3390/molecules30091919 (PMC12073820; doi:10.3390/molecules30091919)
Supplement: Supplementary file 1 [file molecules-30-01919-s001.zip › molecules-3546089-supplementary.pdf]

Table S1. Major components of GCT and GYT (%)

Peak number	Constituents	Content (%, w/w)	
		GCT	GYT
1	GA	0.15±0.00 [a]	0.08±0.01 [b]
2	TB	6.21±0.18 [a]	0.17±0.00 [b]
3	THEO	ND	ND
4	GC	2.37±0.04 [a]	2.05±0.88 [b]
5	CAF	0.05±0.00 [a]	8.44±0.06 [b]
6	EGC	2.67±0.04 [a]	7.94±0.16 [b]
7	CA	10.43±0.20 [a]	2.96±0.04 [b]
8	EC	0.79±0.01 [a]	10.12±0.15 [b]
9	EGCG	2.37±0.09 [a]	25.88±0.04 [b]
10	GCG	38.01±0.98 [a]	2.58±0.02 [b]
11	ECG	1.23±0.07 [a]	19.40±0.13 [b]
12	1,2,4,6-GA-glc	10.25±0.29 [a]	ND
13	CG	0.70±0.29 [a]	0.56±0.01 [b]
14	GC-3,5-diGA	6.60±0.14 [a]	ND
/	Total *cis*-catechin	7.05±0.07 [a]	63.33±0.16 [b]
/	Total *trans*-catechins	51.51±1.51 [a]	8.16±0.83 [b]
/	Total catechins	58.56±1.58 [a]	71.49±0.67 [b]

Different lowercase letters indicate significant differences in the content of the chemical components in two green tea extracts ($p < 0.05$); ND, not detected.

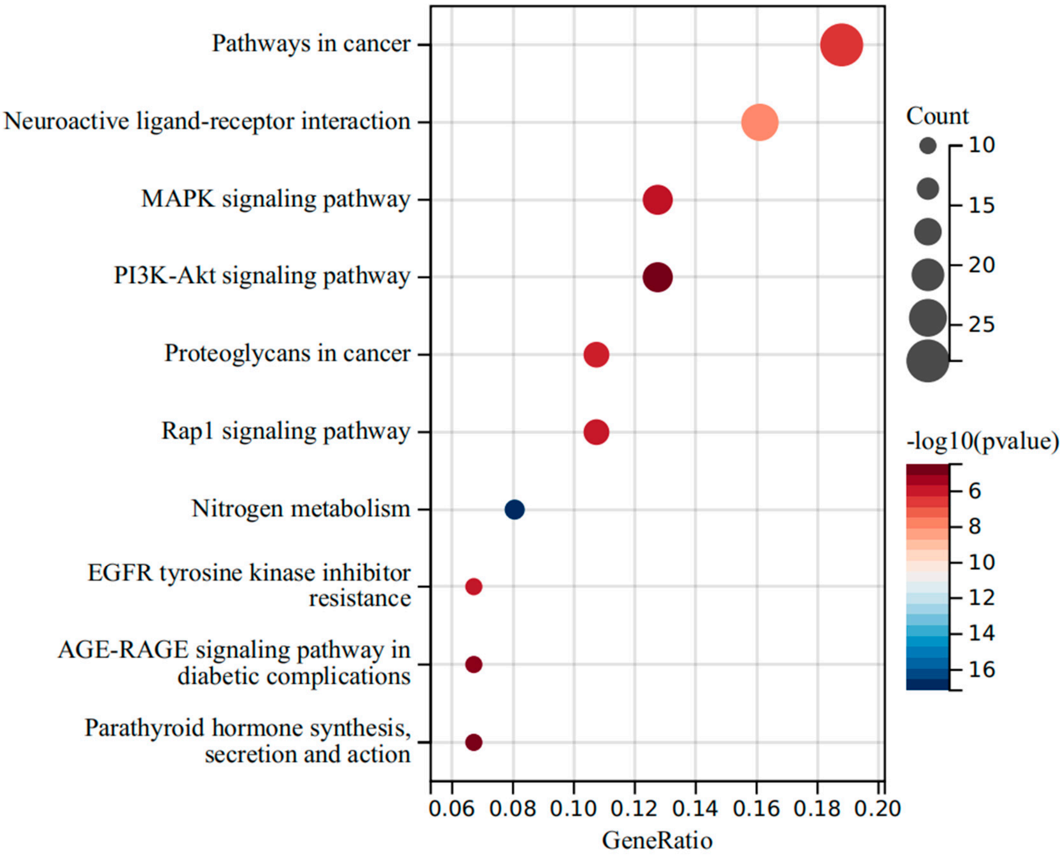

Figure S1. KEGG pathway enrichment analysis of GC-3,5-diGA and 1,2,4,6-GA-glc -associated genes.

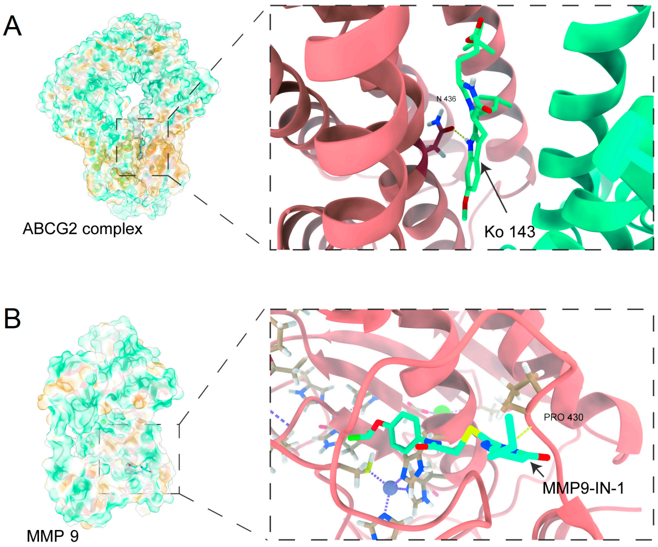

Figure S2. Molecular docking of Ko143 and MMP-9-IN-1 with ABCG2 and MMP9. (A) ABCG2 and GC-3,5-diGA,(B) MMP9 and MMP-9-IN-1.

Table S2. Binding Energy for targets with positive controls.

Target	Inhibitors (positive controls)	Binding Energy (kcal/mol)
ABCG2	Ko143 (CAS: 461054-93-3)	-9.3
MMP9	MMP-9-IN-1 (CAS: 502887-71-0)	-8.8

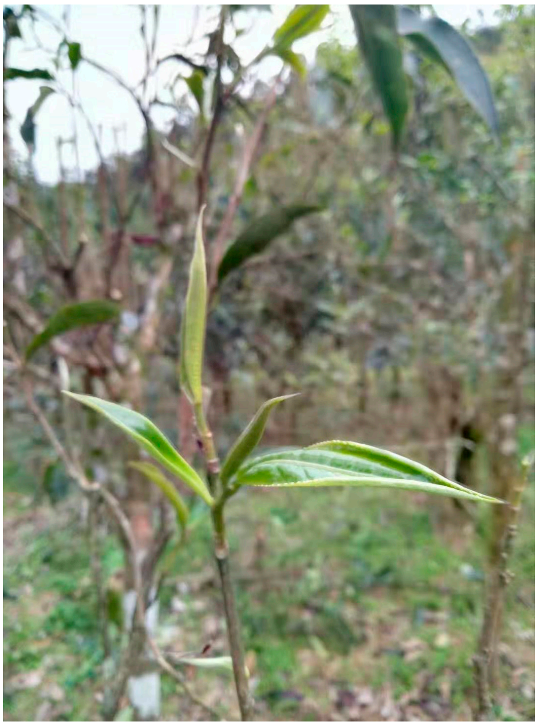

Figure S3. Images of *C. ptilophylla* were collected from Nankun Mountain in spring 2019
